# Network Structure and Community Evolution Online: Behavioral and Emotional Changes in Response to COVID-19

**DOI:** 10.3389/fpubh.2021.813234

**Published:** 2022-01-11

**Authors:** Fan Fang, Tong Wang, Suoyi Tan, Saran Chen, Tao Zhou, Wei Zhang, Qiang Guo, Jianguo Liu, Petter Holme, Xin Lu

**Affiliations:** ^1^College of Systems Engineering, National University of Defense Technology, Changsha, China; ^2^School of Mathematics and Big Data, Foshan University, Foshan, China; ^3^Big Data Research Center, University of Electronic Science and Technology of China, Chengdu, China; ^4^West China Biomedical Big Data Center, West China Hospital, Sichuan University, Chengdu, China; ^5^Research Center of Complex Systems Science, University of Shanghai for Science and Technology, Shanghai, China; ^6^Institute of Accounting and Finance, Shanghai University of Finance and Economics, Shanghai, China; ^7^Tokyo Tech World Hub Research Initiative, Institute of Innovative Research, Tokyo Institute of Technology, Tokyo, Japan

**Keywords:** COVID-19, social behavior, community evolution, sentiment analysis, Sina Weibo

## Abstract

**Background:** The measurement and identification of changes in the social structure in response to an exceptional event like COVID-19 can facilitate a more informed public response to the pandemic and provide fundamental insights on how collective social processes respond to extreme events.

**Objective:** In this study, we built a generalized framework for applying social media data to understand public behavioral and emotional changes in response to COVID-19.

**Methods:** Utilizing a complete dataset of Sina Weibo posts published by users in Wuhan from December 2019 to March 2020, we constructed a time-varying social network of 3.5 million users. In combination with community detection, text analysis, and sentiment analysis, we comprehensively analyzed the evolution of the social network structure, as well as the behavioral and emotional changes across four main stages of Wuhan's experience with the epidemic.

**Results:** The empirical results indicate that almost all network indicators related to the network's size and the frequency of social interactions increased during the outbreak. *The number of unique recipients, average degree*, and *transitivity* increased by 24, 23, and 19% during the severe stage than before the outbreak, respectively. Additionally, the similarity of topics discussed on Weibo increased during the local peak of the epidemic. Most people began discussing the epidemic instead of the more varied cultural topics that dominated early conversations. The number of communities focused on COVID-19 increased by nearly 40 percent of the total number of communities. Finally, we find a statistically significant “rebound effect” by exploring the emotional content of the users' posts through paired sample *t*-test (*P* = 0.003).

**Conclusions:** Following the evolution of the network and community structure can explain how collective social processes changed during the pandemic. These results can provide data-driven insights into the development of public attention during extreme events.

## 1. Introduction

Understanding how social structure and public attention evolve throughout a pandemic can support public policy that is more in line with local understanding and expectations. Additionally, this understanding can provide fundamental insights into how collective social processes evolve, influence participants' emotions, and ultimately change behavior and disease outcomes. In recent years, online social media sites have been increasingly used as agents for understanding the impact of major societal disturbances, such as earthquakes (1, 2), tsunamis (3, 4), and pandemics (5, 6).

Research on major societal disturbances can be divided into three categories: mechanism of information dissemination, early warning and prediction of extreme events, and analysis of public behavioral and emotional changes. Regarding the means of information dissemination, Mendoza et al. (7) examined how rumors and news spread through the Twitter network after the Chile earthquake in 2010 and found that rumors are more likely to be questioned than news. Santosh et al. (8) compared the use of Facebook for Zika-related outreach by the Ministry of Health (MOH) and the National Environmental Agency (NEA). Despite nearly equivalent outreach, MOH's Facebook posts (mainly about situational updates) received more likes and shares, whereas NEA's posts received more comments (mainly about prevention).

Based on Twitter data, Chatfield and Brajawidagda (9) also demonstrated that the tsunami early warning network of the BMKG (the Indonesian Agency for Meteorology, Climatology and Geophysics) Twitter informed 4,102,730 Twitter users of the predicted tsunami within 15 min. Their finding provides clear evidence of the utility of Twitter as a tsunami early warning network. Wu and Cui (10) conducted a hierarchical analysis, not only studied early warning effects of social media at the state level but also explored the relationships between Twitter characteristics, geo-information and damage assessments. Besides, the characteristics of information dissemination on Sina Weibo reported that various types of information about the COVID-19 pandemic were being acquired and exchanged very quickly (11, 12). Bai and Yu (13) focused on negative sentiment microblogging for the early warning and prediction of extreme events. They identified new cases by tracking and predicting trends in the negative emotions of victims. Social media data, such as Facebook and Twitter, can also be used to predict the spread of infectious diseases, including COVID-19 (14, 15), HIV (16, 17), and dengue (18–20). Li et al. (21) described how the peak in Internet searches and social media conversation about the COVID-19 outbreak occurred 10–14 days earlier than the peak of daily incidences in China. The content and sentiment information embedded in these messages can indicate social media users' emotion and behavior, which then provide a useful “sensor” for understanding the public response to exceptional events.

Many researchers have compared the changes in online social platforms before and after disasters to study extreme events' influence on public behavior and emotion. For example, Lu and Brelsford (22) used Twitter data to analyze the dynamics of social networks and the evolution of online communities during the 2011 Japanese earthquake and tsunami. After the earthquake, almost all users increased their online activity. Their behavior of joining or quitting a community was far from random: users tended to stay in their current status. Leveraging the data of Facebook, Phan and Airoldi (23) designed a long-term natural experiment of friendship formation and social dynamics in the aftermath of a natural disaster. Their analysis suggested that affected individuals are more likely to strengthen existing social ties while maintaining the same number of friends as unaffected individuals. Recently, to explore the impact of COVID-19 on public psychology, Li et al. (24) investigated sampled data from 17,865 active Weibo users and found that negative emotions and sensitivity to social risks all increased after the COVID-19 outbreak. Besides, Zhang et al. (25), Yu et al. (26), and Xu et al. (27) all investigated social media posts and described users' emotions and behavior through the content and sentiment information embedded.

Sina Weibo, the Chinese Twitter-like microblogging platform, has become the main channel for obtaining and publishing information about the COVID-19 pandemic. Related literature exploring the role of social media during the pandemic has focused on either the worst periods or the contrast between before and after disasters instead of conducting more detailed segmentation and a comprehensive analysis covering the stage from before the outbreak to the recovery stage. More detailed segmentation of the entire stages can provide us with an opportunity to understand progressive changes in public emotion and behavior from all perspectives. The multiphase evolutionary analytical approach has successfully approved its usefulness to allow decision-makers to take targeted and efficient measures during the life cycle of a public health emergency, serving as a powerful and effective supplement to the offline and pre-social media era methodologies (28). Additionally, given the burstiness and extensiveness of COVID-19, there is an urgent need to understand the social influencing process throughout the development of COVID-19. Specifically, to investigate the impact of extreme events on the behavioral and emotional changes of the public, we construct a time-varying social network based on microblogs from 3.5 million users. We then perform a comprehensive analysis of four aspects of this dataset: (i) text content, (ii) network topology and evolution, (iii) dynamics of users' interests, and (iv) emotional relief. In this way, combined with social media data, we propose a research framework for analyzing public behavioral and emotional changes under extreme events and offer a critical reference for decision making in disease prevention and recovery programs.

## 2. Materials and Methods

### 2.1. Sina Weibo Dataset

During the COVID-19 outbreak, large, sudden onset, and disturbing discussions began spreading extensively online. To understand how the public responded to the epidemic, we collected a comprehensive dataset containing all publicly available Sina Weibo posts from Wuhan. The dataset consists of 42,983,971 microblogs and 3,514,206 unique users and covers the period from December 1, 2019, to March 20, 2020, including key fields: title/content (both the text and emojis in a post), original/forwarding (whether the post is original or forwarded), nickname (used as an identifier for unique users), posting time, original content, authentication type (includes five levels: “gold v,” “orange v,” “blue v,” “talent,” and “ordinary”), etc. In order to protecting users' privacy, userID, postID and part of the dataset are encrypted and cannot be viewed or restored.

According to the epidemic's developing trend and characteristic, we divided the study period into four stages: (1) Before the outbreak, from 00:00 December 1, 2019, to 23:59 December 31, 2019. COVID-19 related words have not appeared on Weibo during this stage. (2) Initial stage, from 00:00 January 1, 2020, to 23:59 January 31, 2020. The number of confirmed cases during this stage (3,215) was less than 7% of the total confirmed cases on March 20, 2020 (50,005). (3) Severe stage, from 00:00 February 1, 2020 to 23:59, March 2, 2020. In this stage, 46,211 new cases were identified. A large jump in new cases during this stage—more than 14,840 in Wuhan—was due to a change in the criteria for counting cases (29). If these diagnostic criteria had been in place during the initial period, then many of these 14,840 cases would likely have been attributed to the pandemic's initial stage. Importantly, these cases were not publicly identified during the initial stage and did not influence public conversations until they were known. (4) Recovery stage, from 00:00 February 19, 2020 to 23:59 March 20, 2020. The number of new cases on February 19, 2020 dropped from 1,552 (February 10, 2020) in the severe stage to 615 and tapered off. On March 18, 2020, no new cases were reported in Hubei Province, and the pressure of the epidemic had been relieved. Public life was also largely back to normal, with school and office attendance at typical levels.

### 2.2. Network Construction

For each of the four stages, we constructed a directed weighted social network of the Weibo users. Let ν_*i*_∈*V*(*i* = 1, 2, ⋯ , *n*) represent a user who sends or receives a microblog in the dataset, and each user is uniquely identified by their nickname. (ν_*i*_, ν_*j*_)∈*E* forms when user *i* directly refers to user *j* or forwards a microblog written by *j*. *W*(ν_*i*_, ν_*j*_) denotes the number of times *i* mentioned *j* in all microblogs during the study period. The network, say *G* = (*V, E*), consists with the node set *V* = {ν_1_, ν_2_, ⋯ , ν_*n*_} and links set *E* = {*e*_1_, *e*_2_, ⋯ , *e*_*m*_}⊆*V* × *V*.

### 2.3. Community Detection

To observe the evolution of community structure during the epidemic, we applied the Infomap algorithm (30) to detect the community structure in the network at four stages. The Infomap algorithm has the flexibility to find community structures for either undirected or directed, weighted or unweighted networks and is found to be among the most effective, reliable and accurate community detection methods (31, 32).

With *map equation* (33) as the objective function, the algorithm applies the random walks (30, 34) to realize clustering. Infomap uses minimum description length (MDL) to measure the quality of detected community results, where a shorter MDL means a more compressed community structure (35). The definition of *map equation* is shown by:


(1) $$\begin{array}{l} L\left(M\right)=q_{↶}H\left(Q\right)+\overset{m}{\underset{i\;=\;1}{\sum }}p_{↻}^{i}H\left(P^{i}\right) \end{array}$$

In (1), where *M* is the set of modules, *q*_↶_ is the sum of the exit probability of each module in the graph, and *H*(*Q*) is the average code length of movements between the modules. $p_{↻}^{i}$ is the stay probability for the random walks in a module *m*, which is equal to the sum of the exit probability and the visit probability of the random walks, and *H*(*P*^*i*^) is the average code length of a module codebook for *m*. *H*(*P*^*i*^) represents the lower bound on the code length of detected community structure *M*.

The general steps of the algorithm are as follows: (1) Treat each node in the network as an independent group. (2) Randomly sample a sequence of nodes. (3) Assign each node to the community where its neighbor node belongs to, and calculate each MDL gain. Assign the community with the largest decline of *L*(*M*) to the node. If it does not drop, the community of the node remains unchanged. (4) Repeat step (3) until the communities become stable (i.e., the MDL change is less than a predefined threshold θ) or the iteration number reaches a user-specified maximum number.

### 2.4. Text Analysis

To identify topics of analysis, we conducted an analysis of theme rivers for each stage in a random sample of 10% *ordinary users'* microblog text through Latent Dirichlet Allocation (LDA) (36). LDA is a generative probabilistic model and a three-level hierarchical Bayesian model, which refers to the word, the topic, and the document.

As a form of unsupervised learning, LDA views documents as bags of words (i.e., the order does not matter). It works by first making a key assumption (called γ). The way a document is generated is by picking a set of topics and then picking a set of words for each topic, which can be represented by


(2) $$\begin{array}{l} P\left(word|document\right)=P\left(word|topic\right)P\left(topic|document\right) \end{array}$$

The process for extracting a topic from a document *m* is as follows. (1) Assume there are *k* topics across all of the documents. (2) Distribute these *k* topics across document *m* (this distribution is known as α and can be symmetric or asymmetric) by assigning each word a topic. (3) For each word *w* in document *m*, assume its topic is wrong, but every other word is assigned the correct topic. (4) Probabilistically assign word *w* a topic based on two criteria: what topics are in document *m* and how many times word *w* has been given a particular topic across all of the documents (this distribution is called β). (5) Repeat this process several times for each document until α and β comply with the assumption γ. In the context of text modeling, the probabilities of topics provide an explicit representation of a document.

### 2.5. Sentiment Analysis

Considering training time and accuracy, we performed sentiment analysis using the Bidirectional Long Short-Term Memory (Bi-LSTM) model (37) to quantify the emotion of the microblog text (More details see Supplementary Table 2). With a score of 0 indicating the most negative emotional content and 1 the most positive. First, we conducted a preliminary analysis for a training dataset with 15,000 posts through a sentiment lexicon and manually labeled the sentiment score with cross-validation [i.e., each post was double-checked by two graduate students (a third student will review the score if there is a large discrepancy between the rates of the two students)]. Then we set the hidden size of the Bi-LSTM layer as 64 and the dropout probability (which will lose some units at random during training) as 0.4. The loss function was chosen as cross-entropy, and the evaluation index was set to the accuracy. We trained the model using Adam (a stochastic gradient descent algorithm for optimizing objective functions) (38) and stopped training when the loss did not decrease. For all datasets, we divided the data into train/validation/test splits using a ratio of 0.8/0.1/0.1. To rule out the effects of different stages, the reliability of the model was verified in a random sample of 10,000 microblogs from each of the four stages, for a total of 40,000. These data were scored manually according to the rating criteria. A comparison of the model results with human-annotated scores reveals that the accuracy rates of the score in the four stages are all above 86%.

## 3. Results

### 3.1. Text Analysis

To understand the overall temporal evolution of the topics dominating online conversations as the epidemic evolved, three kinds of in-depth mining going from the easy to the complex and complicated were implemented by natural language processing. Epidemic-related keywords with high frequency were first analyzed to reveal users' attention toward COVID-19. We then investigated high-frequency keywords covering all microblog texts, obtaining more general information about users' concerns and opinions across all four stages. Third, we described the hidden topics, changing with the epidemic evolution, among *ordinary users* by LDA, which are primarily different from discrete frequency information of the keywords.

#### 3.1.1. Frequency of Epidemic-Related Keywords

**Figure 1:**
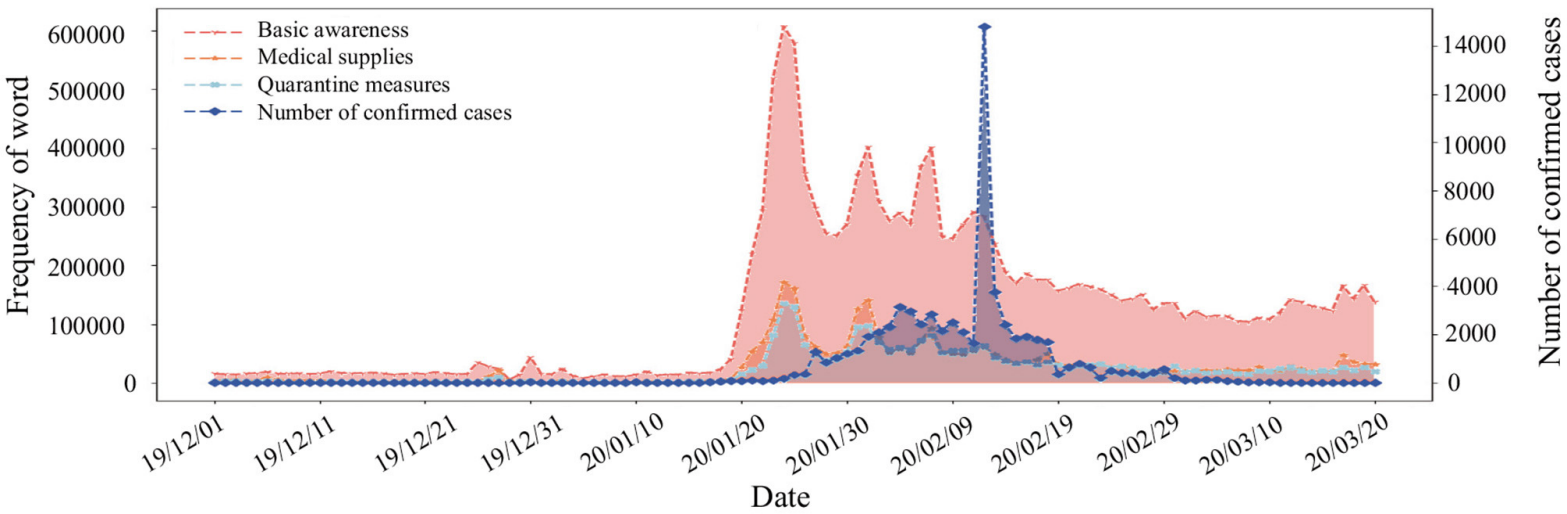
Trend of confirmed cases and frequency of the epidemic-related keywords.

Microblogs about COVID-19 were filtered from the full dataset based on a prior set of keywords related to the epidemic (39, 40) (see Supplementary Textbox 1). After conducting text segmentation for these microblogs, we further ordered keywords of segmentation by the frequency of every keyword. To analyze the relationship between the frequency of different keywords and the evolution of the epidemic, we extracted 55 epidemic-related keywords with a frequency above 4,000 (see Supplementary Table 1). We divided them into three main types: *Basic awareness, Medical supplies*, and *Quarantine measures*. Next, we calculated the number of microblogs containing keywords from the three categories. From Figure 1, we can see that *Basic awareness* is the type with the largest number of microblogs related to the epidemic, which indicates most people's concern primarily focus on *Basic awareness* rather than some awareness deeper like “mask” and “social distance.” Also, the changes in the number of various microblogs have a similar trend with the increase of confirmed cases and a particular role in early warning (41, 42).

#### 3.1.2. High-Frequency Keywords

**Figure 2:**
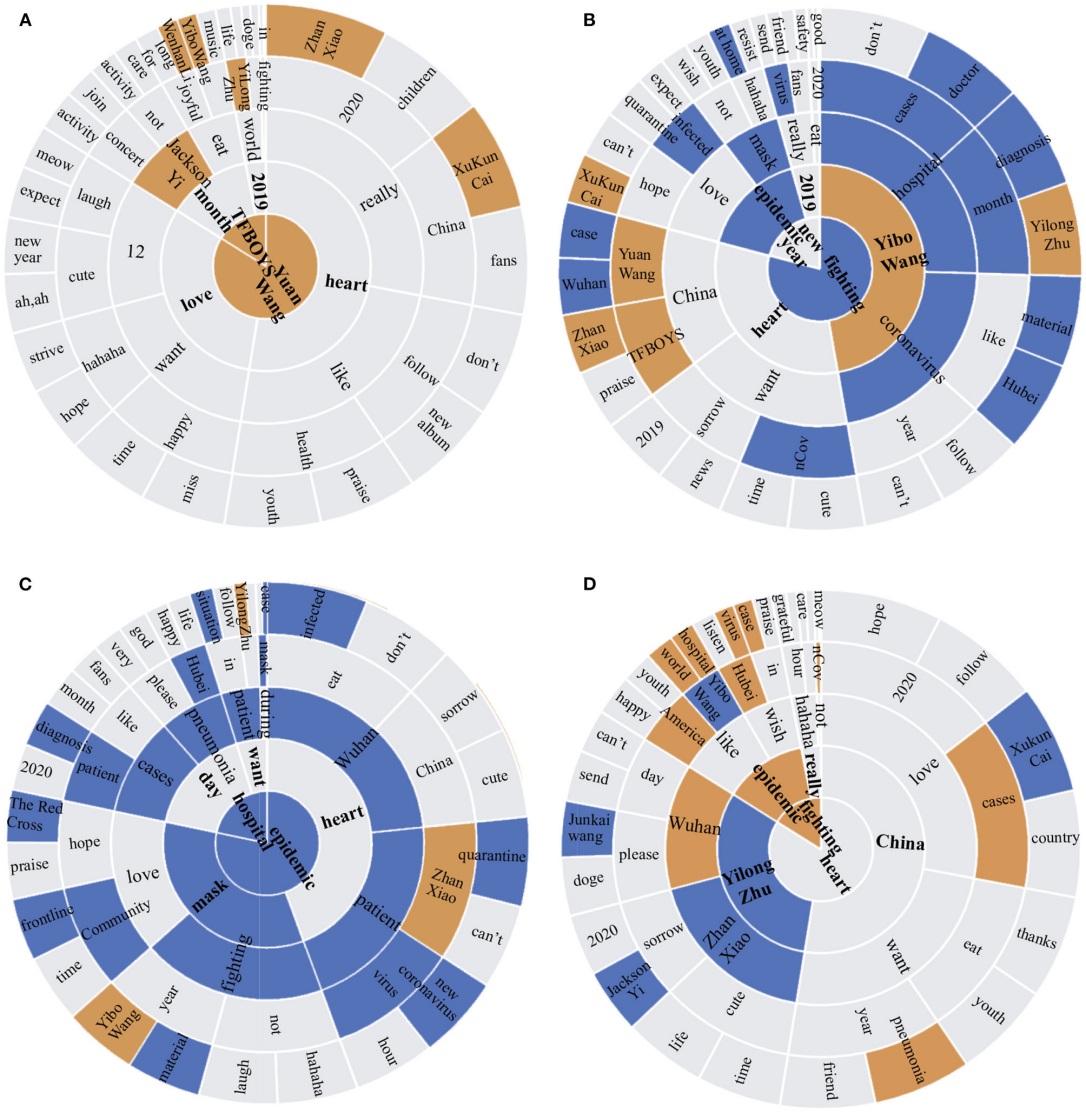
Sunburst plots of Weibo content in four stages: **(A)** Before the outbreak; **(B)** Initial stage; **(C)** Severe stage; **(D)** Recovery stage. The closer a word is to the center of the circle, the higher the frequency of its presence. Moreover, three different topics are distinguished by color, including blue for the epidemic, brown for stars, and gray for social chatter. The non-translated plots are shown in Supplementary Figure 1.

Figure 2 plots the sunburst chart based on the high-frequency keywords extracted from the Weibo content of four stages. In December 2019, most posts focused on popular topics such as entertainment and life when the disease was not yet known. In the initial stage, with the epidemic rapidly developing in Wuhan, words related to COVID-19 began to appear. Although words related to COVID-19 did not occupy a vital position of the word cloud, which meant these words appeared with low frequency, they did have a high proportion on the whole. As time went on, topics related to the epidemic increased, such as “hospitals,” “epidemic,” and “mask,” became the dominant topics in the severe stage. Simultaneously, the word “mask” was relatively close to the center in the severe stage and the recovery stage, which implied that interventions had gathered public understanding and support. In the recovery stage, the appearance of entertainment topics increased, which illustrated that public lives were gradually resuming their normal state. However, epidemic-related words still existed, which might be associated with the foreign epidemic situation.

#### 3.1.3. Topics

**Figure 3:**
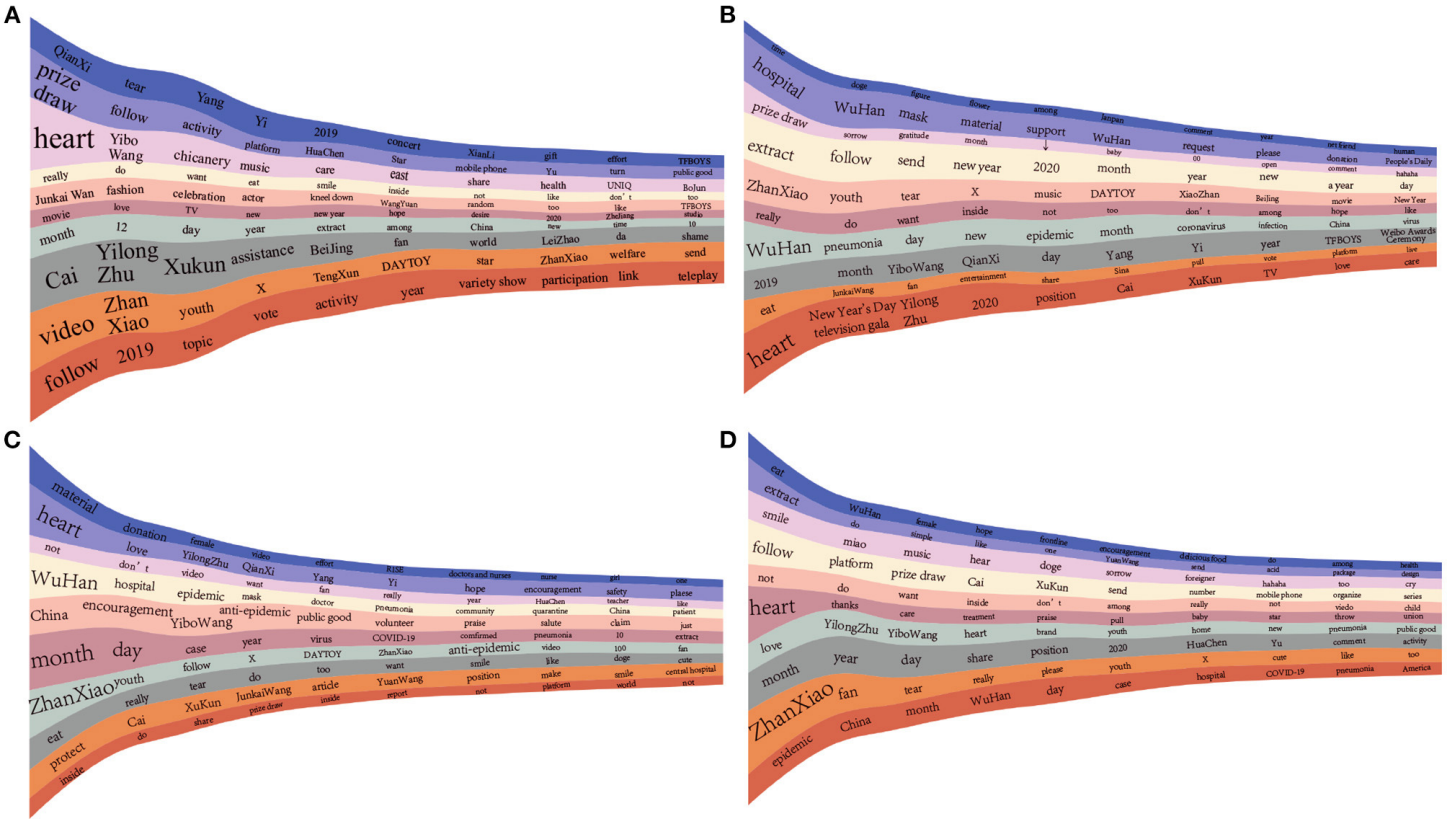
Theme river for *ordinary users* in the four stages. **(A)** Before the outbreak; **(B)** Initial stage; **(C)** Severe stage; **(D)** Recovery stage. The non-translated plots are shown in Supplementary Figure 2.

For *ordinary users*, who account for more than 83% of all types of users, 134,871 of them posted at least one microblog in all of the four stages. We then extracted ten representative theme rivers for each stage in a random sample of 10% *ordinary users*' microblog text because the dataset becomes prohibitively massive. From Figure 3, we can see that all 10 theme rivers were about celebrities, hotly debated issues, and daily topics when the epidemic had not yet taken place. With the epidemic proliferating, two theme rivers related to COVID-19 appeared in the initial stage: “epidemic in Wuhan” and “material support.” In the severe stage, the number of topics pertaining to the epidemic constituted half of all topics, including the growing trend of the epidemic, control measures, and the communities and hospitals' concrete situation. Step by step, the spread was brought under control, and topics of entertainment draw more attraction compared with those of the epidemic. Only one epidemic-related topic, mainly about the number of confirmed cases, remained.

### 3.2. Network Characteristics

**Figure 4:**
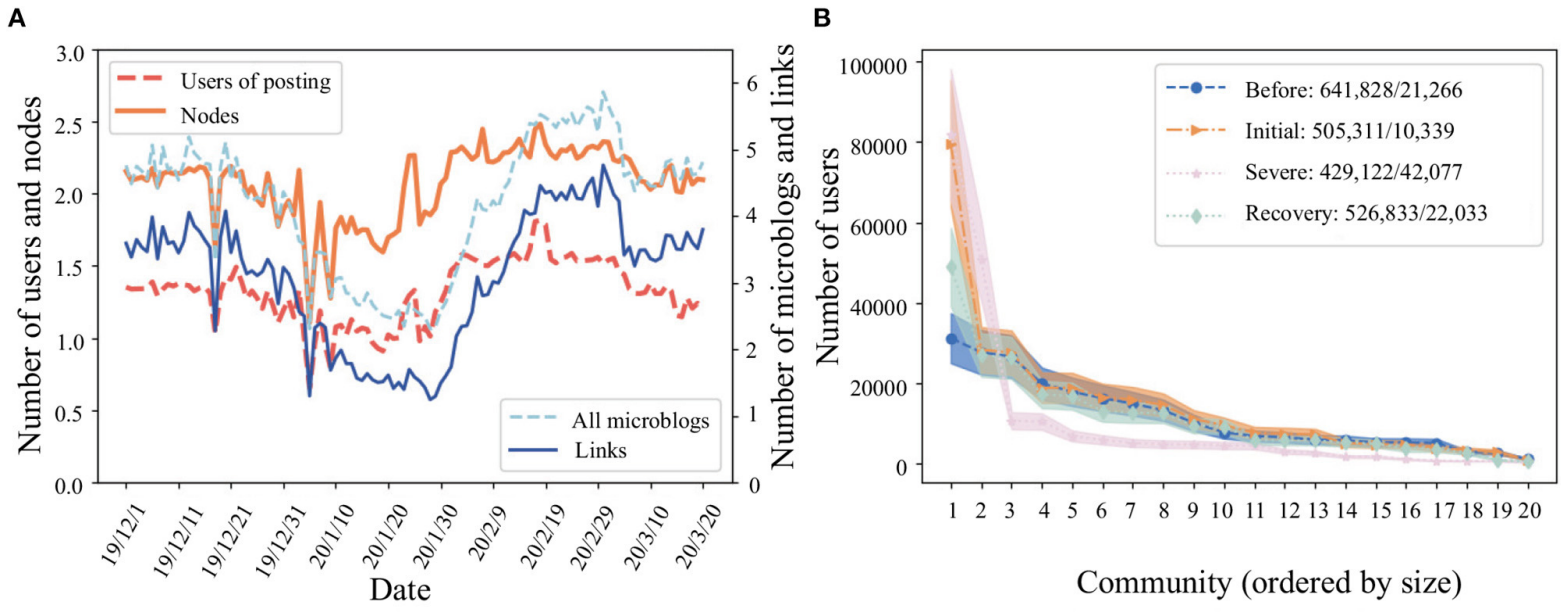
**(A)** Overview of the posting activity. **(B)** Size of the top 20 network communities during the four stages. The number of communities and the number of links between communities are shown before and after “/” in the legend.

COVID-19 affected the topics that Weibo users posted about and was associated with changes in the users' communication networks. We investigated the temporal evolution of the network structure by analyzing the network characteristics of our four stages: before the outbreak, initial, severe, and recovery. By any measure, people were most active on Weibo during the severe stage of the epidemic. In Figure 4A, we show: The number of active users (those who published at least one post), network nodes (all users who are included in the network, including those who did not post themselves but were mentioned by an active user), total posts, and links between users all reached a maximum in the severe stage.

In terms of network interactions, we calculated different indicators, including *the average number of unique recipients* (number of users referred to or forwarded directly by a user), *average degree*, and *transitivity* (43), for each of our four stages. Each of these indicators is larger during the severe and recovery stages than in the initial stage. This finding indicates that Weibo users expanded their active social network to a broader and denser communication network when the epidemic happened. We also note that the distributions of in-degree and out-degree for the four stages are consistent with heavy-tailed, power-law-like distributions, which indicates that the degrees of the vast majority of Weibo users are rather low, while only a small proportion of users interacting with a large number of others; 80% of users have less than 4 links while top users have more than 35,000 links (see Supplementary Figure 3). The increase in network interaction strength is driven by a few large-percentage increases in the number of links for *ordinary users*, rather than extreme increases in the highly connected “gold v,” “orange v,” and “blue v” top users.

### 3.3. Evolution of Network Communities

The substantial change in online interactions for Weibo users leads to a strong variation in the formation of network communities, which divides the network into several groups, within which the connection between nodes are relatively more frequent than the connections between different groups. With the Infomap algorithm, we detected the community structure in the network of four stages (Figure 4B). Although users and communities reduced in total number as the epidemic spread, the links between communities increased significantly. Precisely, most communities followed with interest the same or similar topic during the epidemic. This demonstrated that a high degree of aggregation and similarity exists among the topics of communities. The majority of users belonged to the five largest communities. In the initial and severe stages of the epidemic, conversation in these communities was primarily focused on topics related to the epidemic, occupying 19.5 and 23% of all users, respectively. This result implies that public concern is quickly caught up in emergencies as they happen. Of course, we also note that Wuhan was the global epicenter of the COVID-19 pandemic, and so much of the global news, conversation, and ideas about COVID-19 in this stage arose in Wuhan.

**Figure 5:**
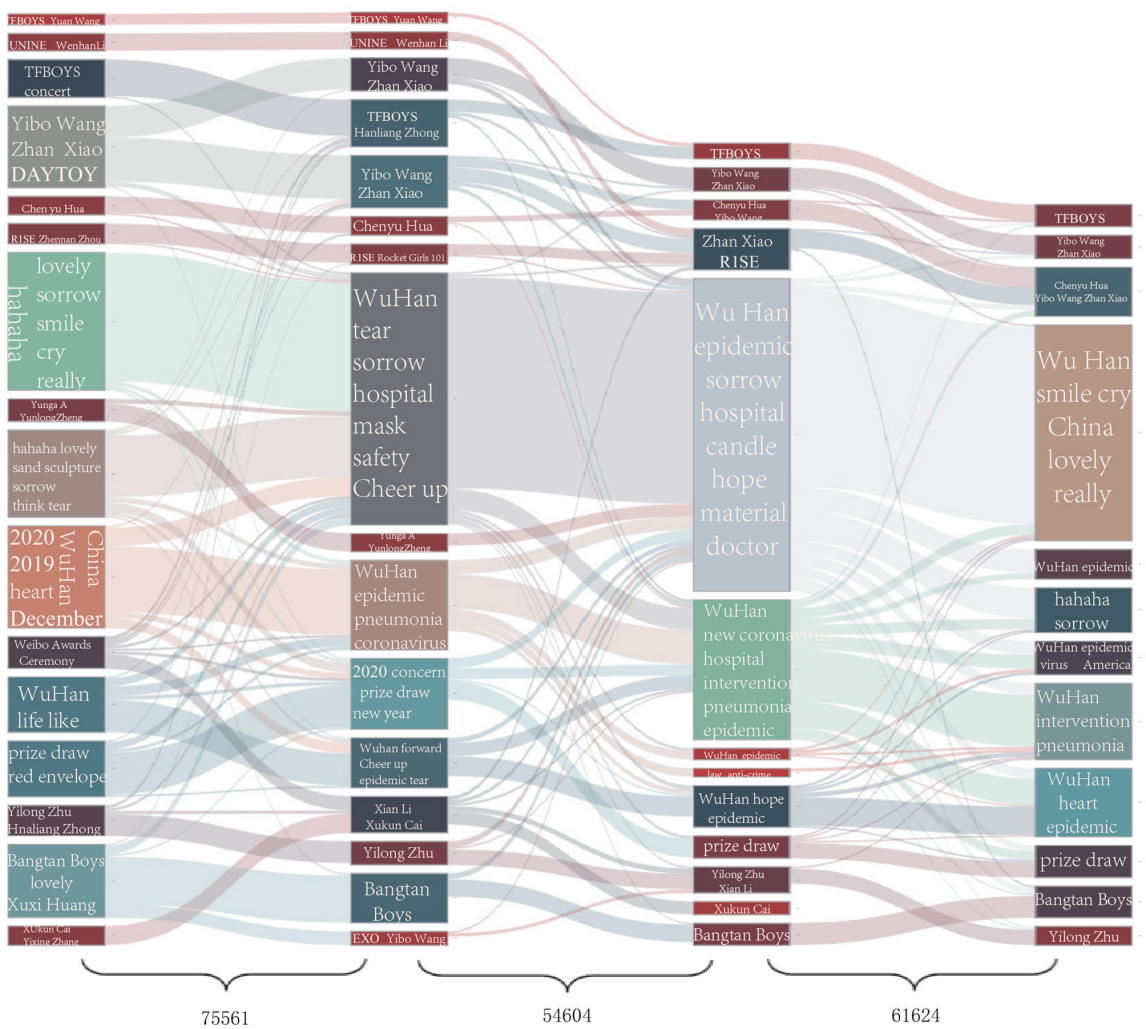
Evolution of network communities during the COVID-19 outbreak in Wuhan. Flows with less than 100 users are filtered, and the non-translated plots are shown in Supplementary Figures 4–8.

Figure 5 shows users' flow among the top 20 communities in four stages with an alluvial diagram. The height of the bars representing communities is proportional to the number of users. We display each community's content with a word cloud in which the font size is proportional to its frequency. The flow of users (i.e., users who transfer from one community to another) between communities at each epidemic stage became more frequent from the initial to the severe stage and from the severe to the recovery stage. Focusing on each community, we observe that the factor that has the most substantial influence on community members' flow is the change of hotly debated issues, such as Weibo Awards Ceremony (44), Shanghai International Musical Festival (45), and COVID-19. Among these issues, the COVID-19 epidemic had the most decisive and most direct impact; movements between other communities seem to be driven by topic similarity. For example, users transitioned between communities whose conversations focused on Yibo Wang and Zhan Xiao—both popular actors. Most significantly, the core topic of public conversation switched from life, entertainment, and cultural events to the domestic epidemic and then to the global pandemic.

Before the COVID-19 outbreak, 18 of the 20 communities discussed topics about life, entertainment, and culture. The remaining communities frequently mentioned the word “Wuhan,” which at that time typically referred to the location of users and was not yet linked to COVID-19. In the initial stage, 3 of the 20 communities' topics closely focused attention on COVID-19 while the rest also mentioned epidemic-related words to a more limited extent; these phrases included words such as “pneumonia,” “masks,” and “hospital,” hinting that public concern about the epidemic was gradually rising. Conversations around life, entertainment, and culture remained prevalent, with 17 of the 20 communities focused on these topics.

In the severe stage, the number of communities focused on COVID-19 increased to eight. Discussions about the epidemic expanded remarkably in communities where the epidemic was not the core focus, reflecting a dramatic and widespread increase in public attention to the epidemic. As the epidemic eased in the recovery phase, five of the 20 communities remained focused on COVID-19, and the topics of conversation again became broader. This suggests that local public attention to the epidemic faded as the epidemic became less dangerous in Wuhan even as it rose worldwide. Notably, topics for some of these communities shifted from focusing on the local epidemic and its associated effects to the pandemic abroad.

In our next analysis, we explored the temporal dynamics of individual users' conversation topics. Some users remained focused on life, entertainment, and cultural events throughout the entire study period, while others switched to epidemic-related topics. In the top 20 communities, with 646,187 users in total, 0.71% of users (4,609) continually discussed entertainment topics, and 2.15% (13,934) instead focused on the epidemic.

### 3.4. Sustainment of User Interests

**Figure 6:**
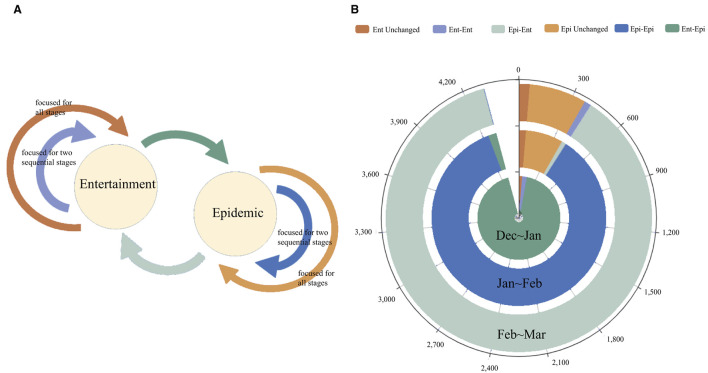
**(A)** Description of user types' evolution and **(B)** evolution of user types in four stages.

The previous analysis implies communities survive substantial social disruption. In this section, we carried out further analysis of the sustainment of users' interests, explored by keywords. Since the *ordinary user* (the user whose authentication type is “ordinary”) is the largest group and the most representative of public behavioral changes, we extracted the original microblogs of 10% of all *ordinary users* for our next research in four stages. To better understand users' behavioral changes, we gathered the users into six clusters using the K-means unsupervised text clustering. We then divided them into two types: those focused on *entertainment and lifestyle* and those focused on *epidemic* based on the frequency of the words they used in their posts. Figure 6 highlights transitions between *entertainment and lifestyle* and *epidemic* across the four stages of analysis. As the epidemic continuously spread, there were varying degrees of flow between the two types of users from January 2020 to March 2020. In the initial stage, users mainly transformed from *entertainment and lifestyle* to *epidemic*, owing to the COVID-19's sudden outbreak. As the epidemics subsided, the flow shifted from *epidemic* back to *entertainment and lifestyle* in the recovery stage. In particular, the flow between the two types of users was relatively stable in the severe stage affected by the prevailing epidemic. Although most users' behavior changed over the tendency of the epidemic, concern about a specific topic from a certain percentage of users remained constant, independent of the events of the day. In particular, stable users for the type of *entertainment and lifestyle* and the type of *epidemic* accounted for 1.4 and 7.3% of the total users, respectively. Furthermore, the evolution of users' interests also affects their emotions. Based on sentiment analysis for different users, we find that the sentiment score of entertainment discussants is generally higher than epidemic discussants. This is true independent of whether users are stable (continuously focusing on *epidemic* or *entertainment and lifestyle*) or dynamic users (whose interest kept changing between *epidemic* and *entertainment and lifestyle*). Additionally, dynamic users (the average variance is 0.01275) present stronger mood swings than stable users (the average variance is 0.01182).

### 3.5. Emotional Relief

**Figure 7:**
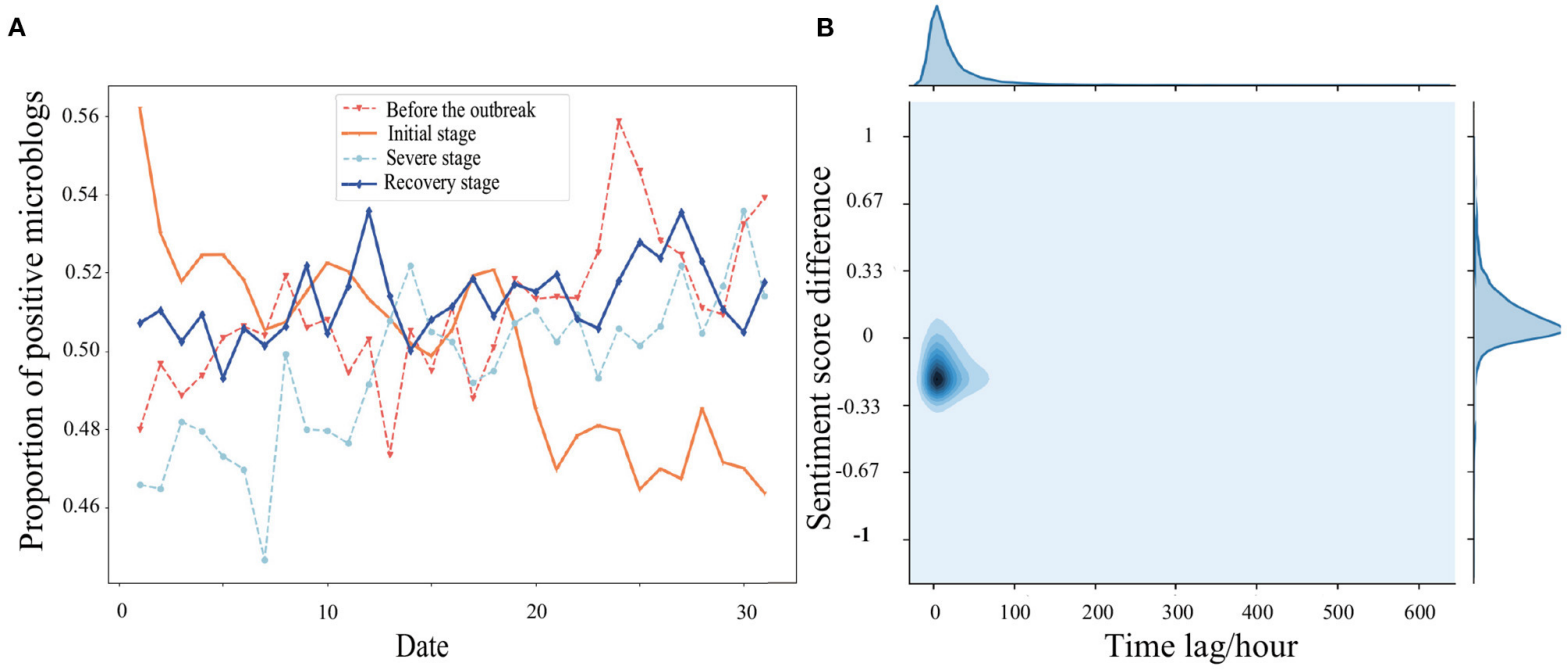
**(A)** Proportion of positive microblogs in four stages and **(B)** correlation KDE map of sentiment score difference and time lag. The x-axis expresses the date of each stage chronologically, for example, for the severe stage it is from February 1, 2020 to March 2, 2020.

The uncertainty of COVID-19 both threatens people's physical health and influences their mental health and emotional state (46, 47). According to the behavioral immune system (48), the fear, uncertainty, and lifestyle disruptions that COVID-19 brought out have caused dramatic psychological consequences for many people, even those not directly harmed by the disease. Related research showed an established correlation between mental health and social media content, and social media can be applied to examine people's emotional change (49, 50). Using the Bi-LSTM model (37), we analyzed the emotional changes of *ordinary users* in four stages (Figure 7A). In addition to the outbreak's initial stage, the proportion of positive microblogs in the other three stages underwent a gradual uptrend. Even in the initial stage of the epidemic, we see signs of emotional recovery after the nadir of January 25, 2020. Relevant studies have demonstrated that negative emotions will be relieved to a certain extent after complaints are made on social platforms or face-to-face (51, 52), prompting us to consider the underlying causes of mood recovery. *Ordinary users* with a weighted out-degree ranking of 10–90% were selected, about 1.7 million, to verify if there is an emotional relief mechanism for Wuhan users' posting behavior. Afterward, sentiment analysis and paired sample *t*-test were performed to examine the differences in sentiment scores between the last microblog and the second microblog. The emotional relief effect is demonstrated to be significant (*P* = 0.003), which means the negative microblog scores posted by *ordinary users* are lower than the next one.

Psychologists also point out that time can heal mental wounds and release negative emotions (53). To rule out this possibility and ensure that *ordinary users'* blogging behavior brings them emotional relief, we made a correlation test for time-interval and sentiment scores. The result is shown in Figure 7B. The coefficient correlations between the sentiment score difference and the time interval of two microblogs posted by the same user are all below 0.1, indicating no direct relationship. A further experiment about the emotional relief effect in four stages suggests that the relief effect's proportions reached over 80% for every stage. This result implies that cathartic blogging causes emotional relief rather than the lapse of time.

## 4. Discussion

This section, details how our understanding of behavioral and emotional changes under extreme events could be augmented by studying their relationship between media effects, domestic and international situations, and government agencies. Furthermore, we propose public health responses according to it, which can help the government and policymakers monitor public response, identify public needs as early as possible, and improve decision-making in implementing control measures (54).

### 4.1. Behavioral and Emotional Changes Under Extreme Events

This paper constructed a generalized framework to track public response and attention to the COVID-19 epidemic, demonstrating a comprehensive and systematic analysis of the individual and population levels.

By following network characteristics and online communities' evolution at the population level, we reveal novel insights about the behavioral and emotional changes evolving with the epidemic. First, almost all network characteristics indicate more frequent online activities and more extensive social networks in the severe stage. This is partly because the government's disease control measures and the public's self-protection awareness led offline communications to shift online for extended periods (29). The local lockdown was active from January 23, 2020, concurrent with the severe stage. Second, the frequent keywords of communities in the severe stage contained more details about the epidemic's time and location (see Supplementary Figure 7), which demonstrated new information about COVID-19 transmitting through individuals' social networks and close public attention and anxiety to the latest trend of the epidemic. Simultaneously, interventions represented by “mask,” “stay at home,” “quarantine,” and “supplies” have attracted great public attention and increased the scale of COVID-19 related discourse (see Supplementary Figure 1). Third, as a significant finding of capturing users' emotional changes on the population level, the “rebound effect” showed that posting a highly negative microblog could relieve the user's negative emotions in our analysis. Social media supplies troubled people with a free space where they may share worries and other expressions of a bad mood, which helps relieve negative emotions stirred by disasters and heighten well-being (55).

As for the individual level, this paper explored the sustainment of user interests. A majority of people presented consistent behaviors with the epidemic development. However, due to various factors such as culture and background, there are 8.7% stable users, including the type of *entertainment and lifestyle* and the type of *epidemic*, whose interests are not affected by the epidemic or other users. Although both of them focus on one same topic continuously, there are different underlying reasons. Based on various criteria like subjective awareness, visibility and the perception of specific social roles (56), users who steadily follow the entertainment get their way without being affected by the COVID-19 situation both at home and abroad. While for users steadily follow the epidemic, although the local epidemic had gradually subsided in the recovery stage, some users still paid attention to the COVID-19 abroad. One cause may be that the public in Wuhan generated enormous empathy for people abroad who were experiencing the same infectious disease (57). Some empathy-related keywords appear in the word cloud of the recovery stage, such as pathetic, fighting, cry, sadness and candle (an emoji for prayer) (see Supplementary Figure 8).

While Sina Weibo exists as the largest Twitter-like social media in China and has shown of full potential for the public health decision-making via mining of the massive posts produced online, it differs from other social media platforms, e.g., Twitter or Facebook, such that there is no political polarization. It is worth noting that the latter had been found to present polarized network structure and community evolution (58, 59). The health- and politics-related discussion received broad attention during the pandemic, and some debates even became increasingly politicized as the pandemic unfolded.

Basically, these analyses show how public concern responded to breaking events and the unfolding of the COVID-19 epidemic into a global pandemic, which would reshape behavior for billions of people globally over the next year.

### 4.2. Social Media and Public Health Responses

Through our inductive examination of how social media could be utilized to fight against COVID-19, many kinds of significant information work from this crisis are remarkably suggestive for public health responses, particularly in support of psychological counseling and emergency responses.

First of all, social media should receive frequent usage for psychological intervention, considering the social distancing during the epidemic and the advantage of immediacy compared to conventional media (60). Similar to (61) and (62), when the public suffers from severe depression, the government could implement timely emotional consultation with the release of information in social media. However, considering the “rebound effect” finding, interventions to negative emotions of the public could be held until many of the users have vented their post-traumatic stress (63), depression, anger, and anxiety online (64). In this manner, resources could be saved and targeted to where they are most needed. What's more, social media analytics could benefit government authorities in public emotional intervention by capturing public perceptions and extracting relevant messages regarding the COVID-19 pandemic (49, 65, 66). For instance, the structural evolution of social networks could be used by risk communication specialists, who should be more active on social media during the peak of interest and have the greatest reach at that time. Meanwhile, learning users' main focus or threads through social media analytics, such as masks and other medical supplies, could also help governments better communicate with the public and translate public health needs into practice to create targeted measures to prevent and control the spread of the epidemic. More importantly, social media offers an effective way of problem feedback that enhances government management's openness and transparency (65).

### 4.3. Limitation and Future Work

There are several limitations of this study that can inform future research. First, a significant limitation is the completeness and credibility of the dataset. Considering the freedom for users to delete posts, some deleted microblogs are missing. Although some misinformation and rumors have been observed to spread across social media (67), we did not detect and reduce misinformation. Second, users' behavior patterns are different due to identity variations (68). So it will be interesting to separate professionals/organizations who primarily disseminate information and “information consumers” which will have more genie discussions among themselves, e.g., friends, colleagues, and family members.

## 5. Conclusions

Our study built a generalized framework for incorporating social media data into understanding the rise and fell of public attention to COVID-19 with a complete dataset of Sina Weibo published in Wuhan. By investigating the network structures and the evolution of online communities in four stages, we see that almost all network characteristics related to the scale of the network and social communication range (e.g., *numbers of edges and nodes, average degree*, and *transitivity*) increased. The result of community detected likewise indicates the extent to which focal topics of conversation in Weibo communities shifted toward the COVID-19 outbreak as the outbreak intensified and then fell back upon more typical conversation topics as the epidemic receded. Conversation topics demonstrated a high degree of aggregation and similarity during the epidemic, and the COVID-19 outbreak was the most crucial factor driving the flow of users among communities. The evolution of community structure and users' attention showed sustained interest in COVID-19 for 2.15% (community structure) / 7.3% (users' attention) share of users. Finally, our results suggest that Weibo posts may provide a meaningful venue for emotional release. The epidemic is not a happy topic. However, it appears that writing a highly negative Weibo post can relieve the user's negative emotions, as expressed in subsequent posts. These results provide data-driven insights to understand public attention and behavioral and emotional changes in response to COVID-19. Exploring the public's behavioral and emotional changes under extreme events is critical for disaster response and recovery. This finding facilitates an empirical understanding of the relationship between attention, behavior, and public health outcomes. Additionally, it is useful for understanding the current epidemic and may promote better public health responses in subsequent public health emergencies.

## Data Availability Statement

The original contributions presented in the study are included in the article/Supplementary Materials, further inquiries can be directed to the corresponding author/s.

## Author Contributions

XL, ST, and PH contributed to conception and design of the study. SC and TZ organized the database. FF and TW performed the statistical analysis and wrote the first draft of the manuscript. XL and JL supervised and validated the project. FF, XL, and QG revised the manuscript. All authors contributed to manuscript revision, read, and approved the submitted version.

## Funding

This study was supported by the National Natural Science Foundation of China (72025405, 91846301, 82041020, 71771213, 71901067, 11975071, 72171150 and 61773248), the Sichuan Science and Technology Plan Project (2020YFS0007), the Hunan Science and Technology Plan Project (2020JJ4673 and 2020TP1013), and the Major Program of National Fund of Philosophy and Social Science of China (18ZDA088 and 20ZDA060). PH received financial support from JSPS KAKENHI (JP 18H01655).

## Conflict of Interest

The authors declare that the research was conducted in the absence of any commercial or financial relationships that could be construed as a potential conflict of interest.

## Publisher's Note

All claims expressed in this article are solely those of the authors and do not necessarily represent those of their affiliated organizations, or those of the publisher, the editors and the reviewers. Any product that may be evaluated in this article, or claim that may be made by its manufacturer, is not guaranteed or endorsed by the publisher.
