# Supplementary material for: Network Structure and Community Evolution Online: Behavioral and Emotional Changes in Response to COVID-19
Source: Front Public Health. 2022 Jan 11;9:813234. doi: 10.3389/fpubh.2021.813234 (PMC8787074; doi:10.3389/fpubh.2021.813234)
Supplement: Supplementary file 1 [file Data_Sheet_1.pdf]

Supplementary Material

1 THE PRIOR SET OF EPIDEMIC-RELATED KEYWORDS USED TO FILTER WEIBO POSTS AND HIGH-FREQUENCY EPIDEMIC-RELATED KEYWORDS AFTER FILTERING

Textbox S1. The prior set of epidemic-related keywords used to filter Weibo posts.

hospital, doctor, nurse, director, medical workers, infection, diagnosis, suspected, patient, case, family, mild, severe, fatal, symptoms, dyspnea, high fever, fever, cough, droplet, cold, pneumonia, flu, SARS, Atypical Pneumonia, virus, Coronavirus virus, unknown, protect, immunity, infection, spread, incubative period, Disease control, Health Committee, HC (Health Committee), CDC, World Health Organization, WHO (World Health Organization), WHO

Table S1. High-frequency epidemic-related keywords after filtering.

Type of keywords	Keyword list
Basic awareness	Wuhan coronavirus, epidemic, pneumonia, New Coronavirus, Wenliang Li, material, Cases, Lanjuan Li, game, sterilize, SARS, flu, fever, antiepidemic, Huanggang City, virus, diagnosis, Nanshan Zhong, Wuhan City, infection, prevention and control, Hubei, patient, contain the outbreak, Wenhong Zhang, discharge from hospital, National Health Commission, Huanggang City, wild animal, treat, medical, suspected case
Medical supplies	mask, protective suit, medical workers, the Red Cross, medical team, nurse, test kit, bed, doctor and nurse, paramedic, doctor, CT
Quarantine measures	quarantine, lockdown, mobile cabin hospital, Union Medical, the god of fire, Central Hospital, Wuhan Union Medical College Hospital, hospital, community

2 EVALUATION OF MODEL PERFORMANCE

In order to select the appropriate model, we have built mainstream machine learning and deep learning sentiment analysis frameworks based on microblog datasets, including Logistic Regression (LR), Gradient Boosting Decision Tree (GBDT), Bidirectional Long Short-Term Memory (Bi-LSTM), Bidirectional Encoder Representations from Transformers (BERT), etc. The evaluation of the models is shown in **Table S2**. Since the categorization of emotions in this paper is rather detailed, i.e., we used eleven categories rather than the typical three categories, strict accuracy evaluation of the models is relatively low. However, it should be noted that the accuracy of sentiment analysis is not the main task of this paper. We only observe the approximate range of users' emotions through sentiment analysis. Therefore, we also evaluated the model accuracy with the criterion that the error between predicted and actual labels is within 0.1. In general, BERT achieves the best classification performance, but its training time far exceeds that of other models, so it is not suitable for such a massive dataset. In contrast, with a shorter training time, Bi-LSTM achieves reasonable classification accuracy with acceptable performance loss.

Table S2. Evaluation of model performance.

Evaluation index / Model	Accuracy	F1-Score	Accuracy(0.1)
LR	0.38	0.18	0.70
GBDT	0.40	0.16	0.72
Bi-LSTM	0.46	0.27	0.72
BERT	0.50	0.27	0.76

3 THE NON-TRANSLATED SUNBURST OF WEIBO CONTENT IN FOUR STAGES

We extract the high-frequency keywords of the Weibo content in four stages and plot the sunburst chart, as shown in **Figure S1**. The closer the word is to the center of the circle, the higher the frequency of it.

4 THE NON-TRANSLATED THEME RIVER FOR *ORIDINARY USERS* IN FOUR STAGES

Because the complete dataset becomes prohibitively massive for LDA-based topic analysis, we extracted ten representative theme rivers for each stage with a random sample of 10% *ordinary users'* microblog text. The non-translated theme rivers are shown in **Figure S2**.

5 USER DEGREE DISTRIBUTION IN FOUR STAGES

In **Figure S3**, we present both the in-degree and out-degree distribution for users in the online social network for the four stages.

6 NON-TRANSLATED ALLUVIAL DIAGRAM OF COMMUNITY EVOLUTION IN FOUR STAGES

With Infomap algorithm, we detected the community structure in the network at four stages. **Figure S4** shows the high-frequency keywords and the flow of users among the top 20 communities detected in four stages with an alluvial diagram. The height of a community is proportional to the number of users in it, and the content of each community is shown with a word cloud in which the size of the font is proportional to its frequency.

7 WORD CLOUDS OF EACH COMMUNITY FOR FOUR STAGES (NON-TRANSLATED)

We plot word clouds of each community for four stages, as shown in **Figure S5 – Figure S8**.

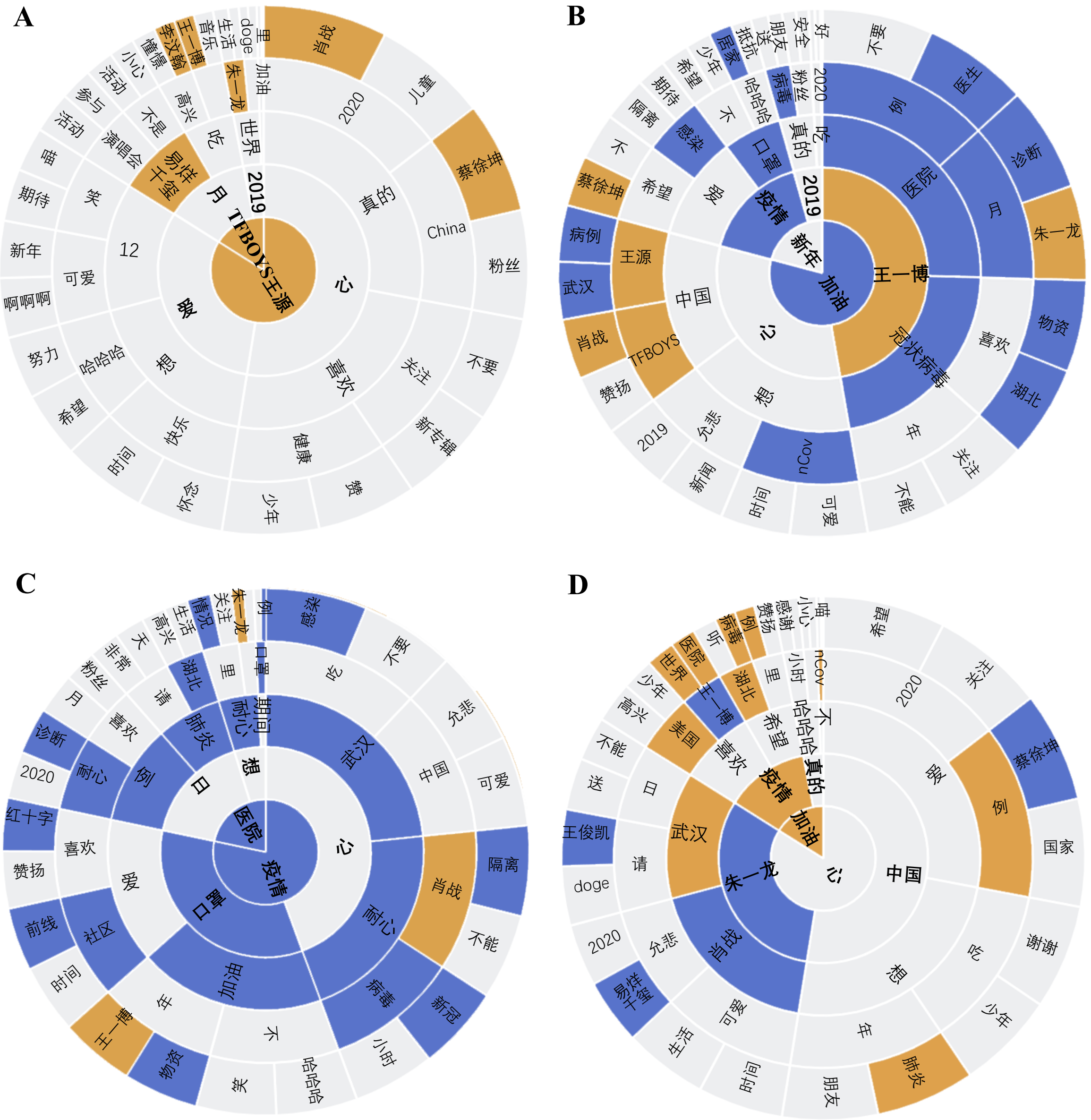

Figure S1. The non-translated sunburst of Weibo content in four stages. **(A)** Before the outbreak; **(B)** Initial stage; **(C)** Severe stage; **(D)** Recovery stage. Moreover, three different topics are distinguished by color, including blue for the epidemic, brown for stars, and grey for social chatter.

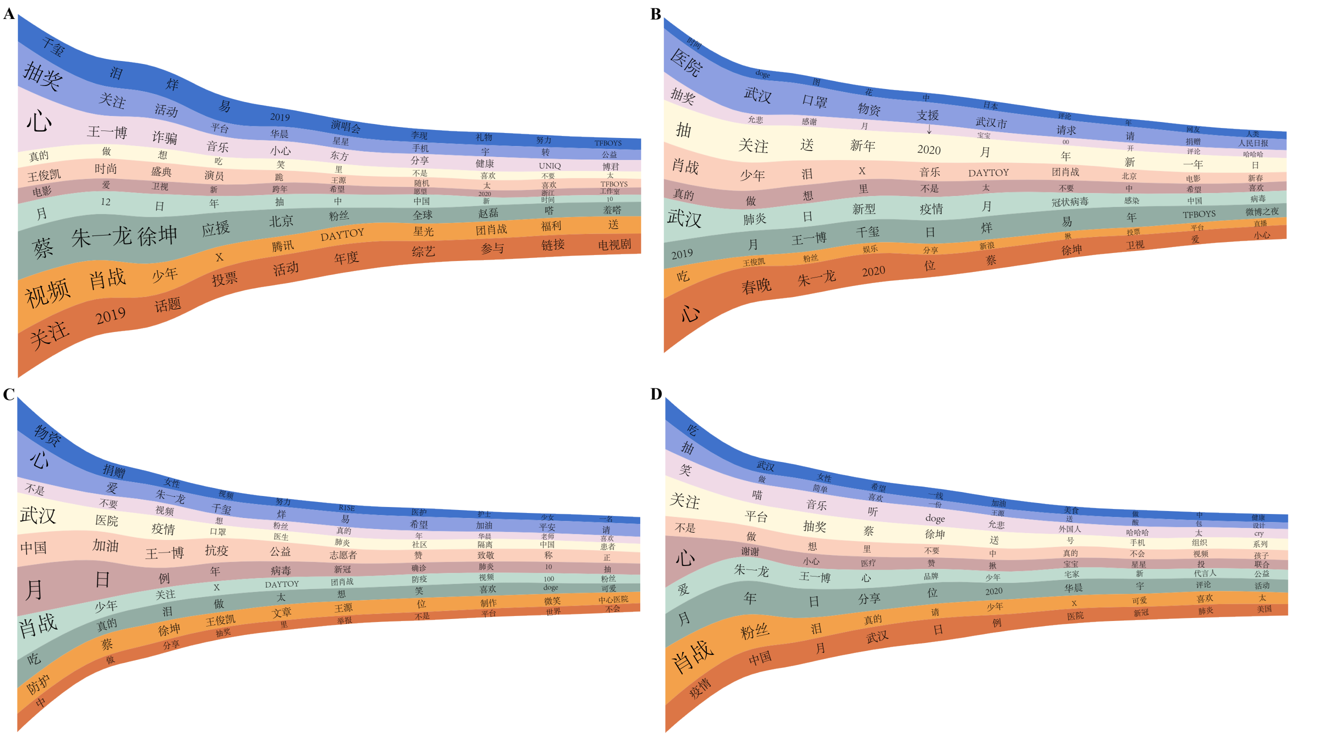

Figure S2. The non-translated theme river for *ordinary users* in four stages. **(A)** Before the outbreak; **(B)** Initial stage; **(C)** Severe stage; **(D)** Recovery stage.

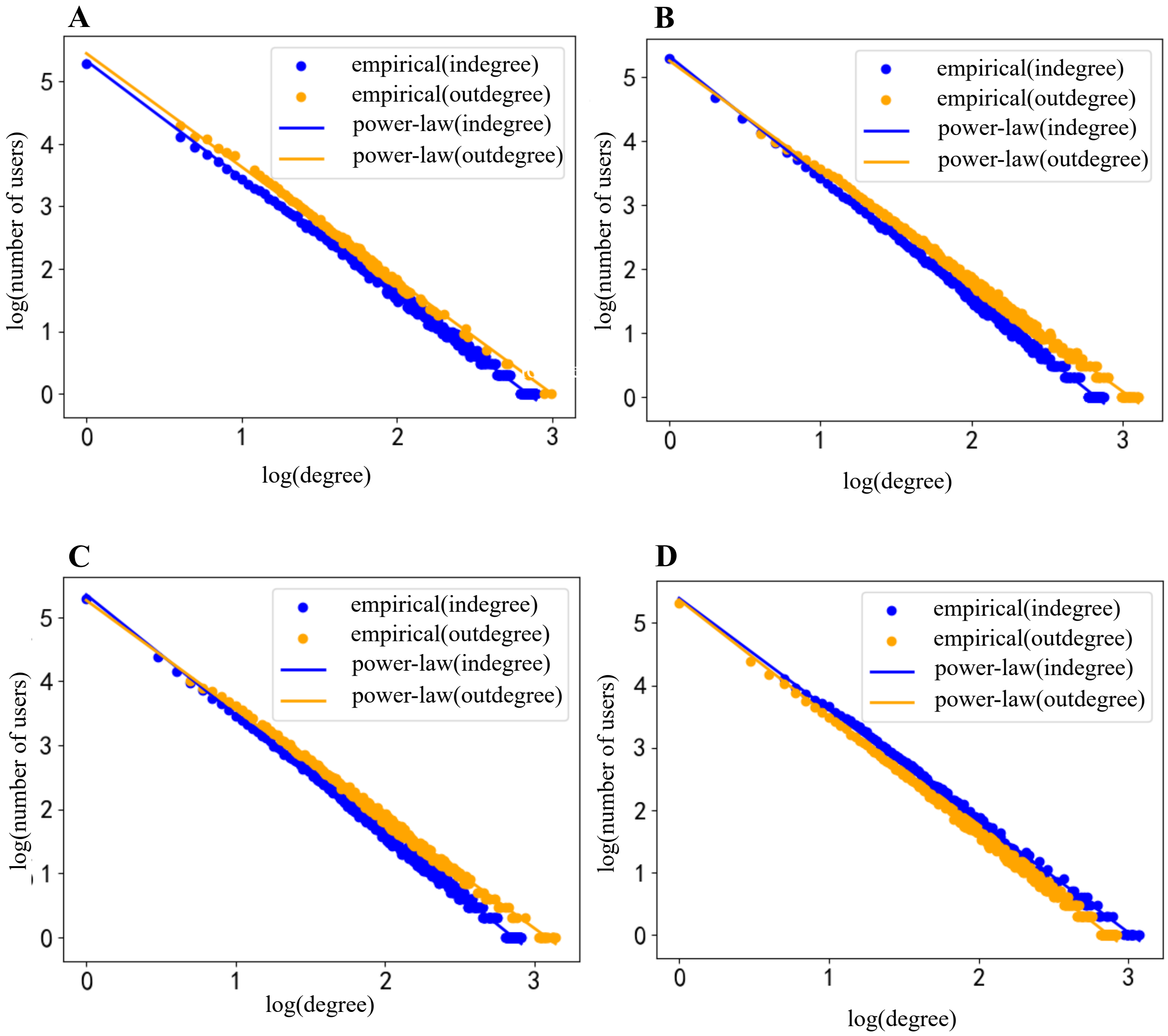

Figure S3. User degree distribution in four stages. **(A)** Before the outbreak; **(B)** Initial stage; **(C)** Severe stage; **(D)** Recovery stage.

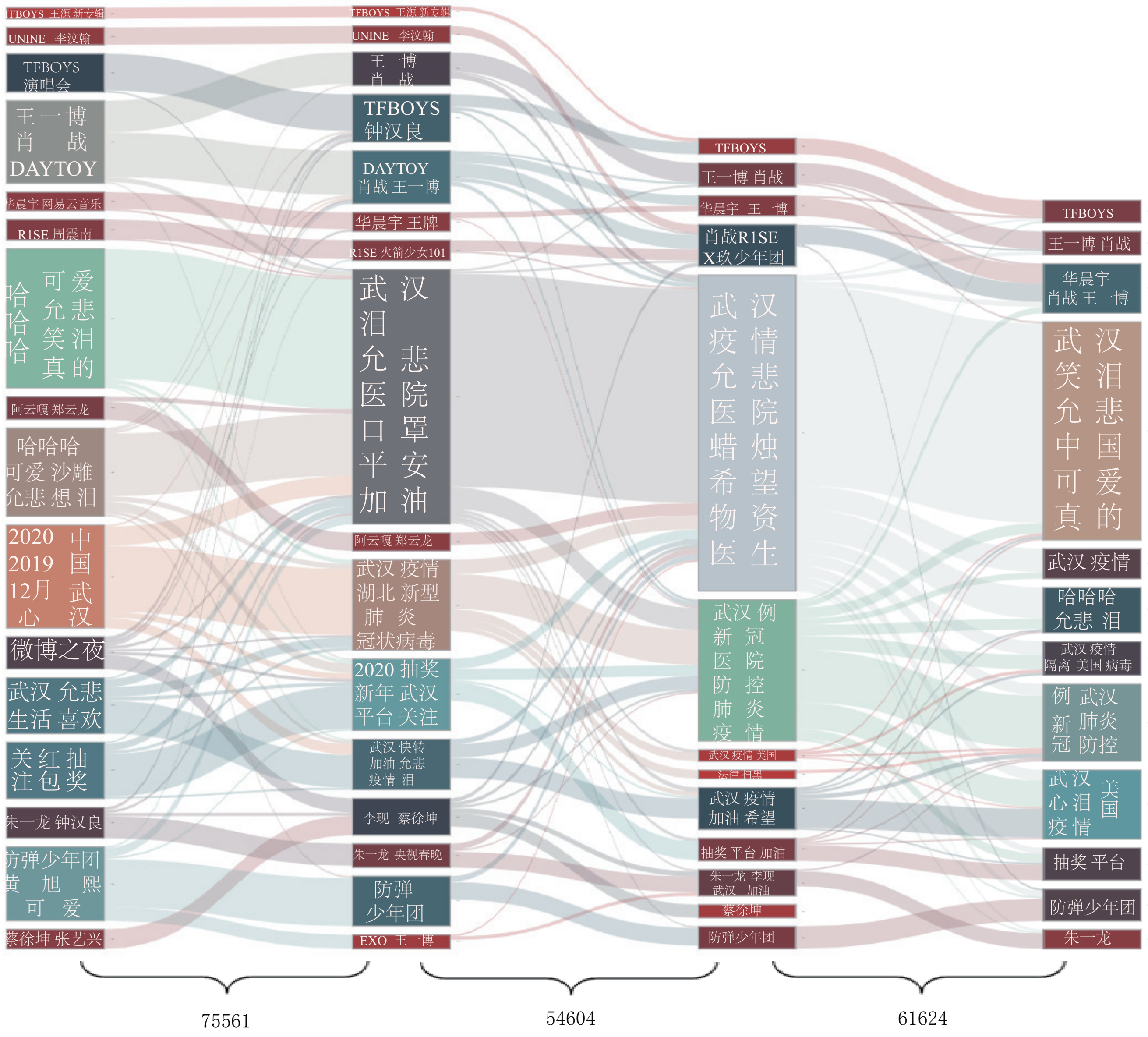

Figure S4. Non-translated alluvial diagram of community evolution in four stages. Flows with less than 100 users are filtered.

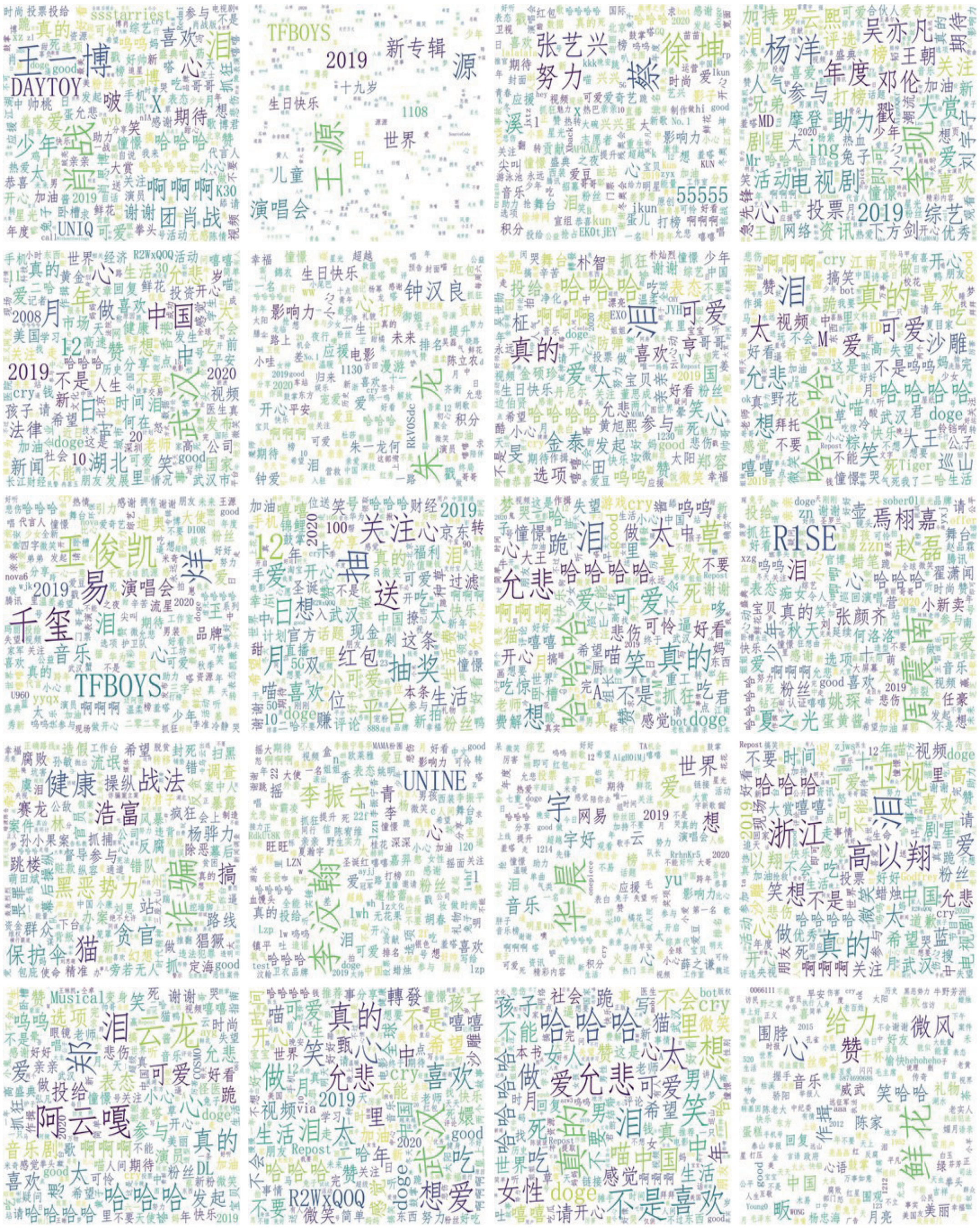

Figure S5. Word cloud of each community before the outbreak.

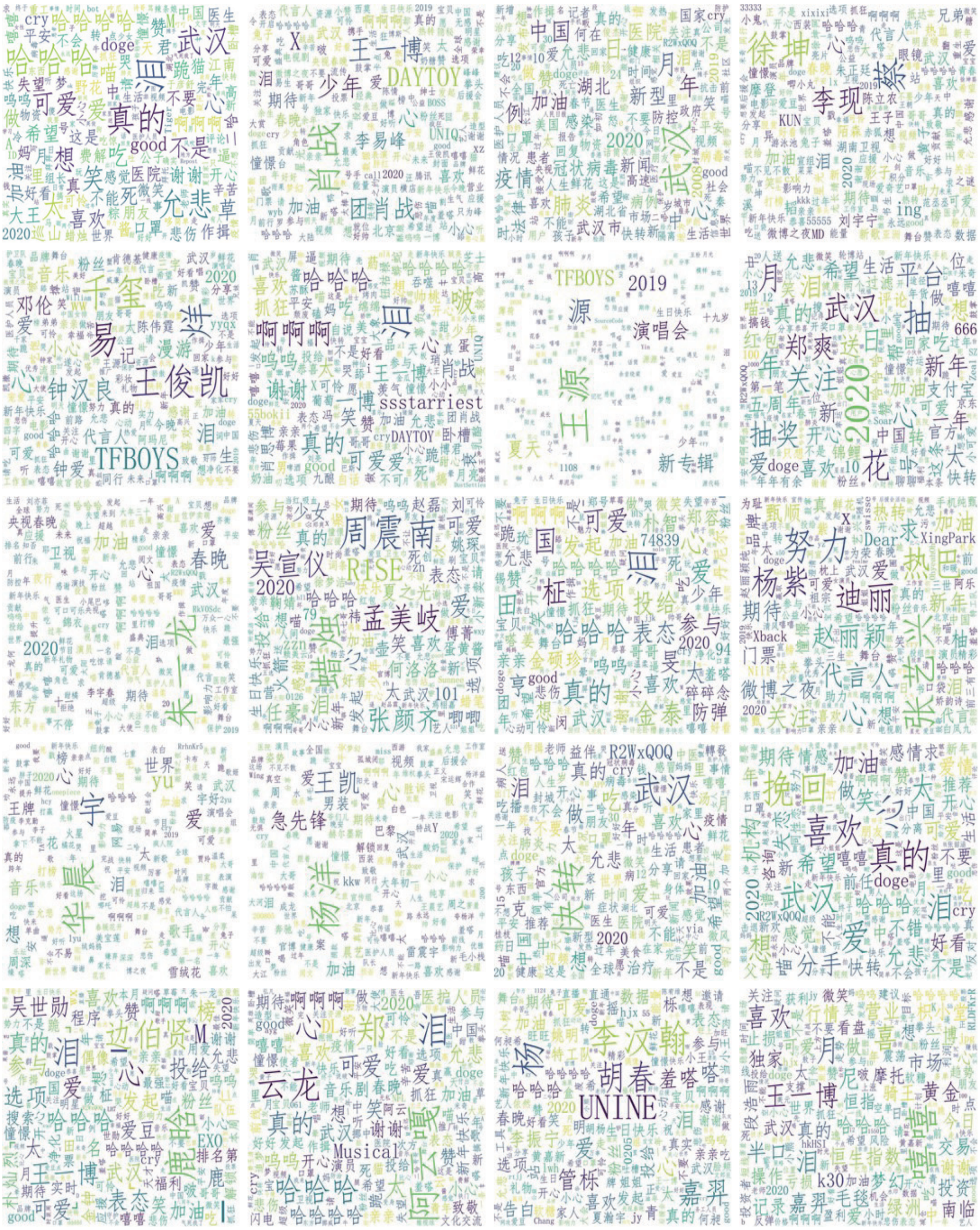

Figure S6. Word cloud of each community in the initial stage.

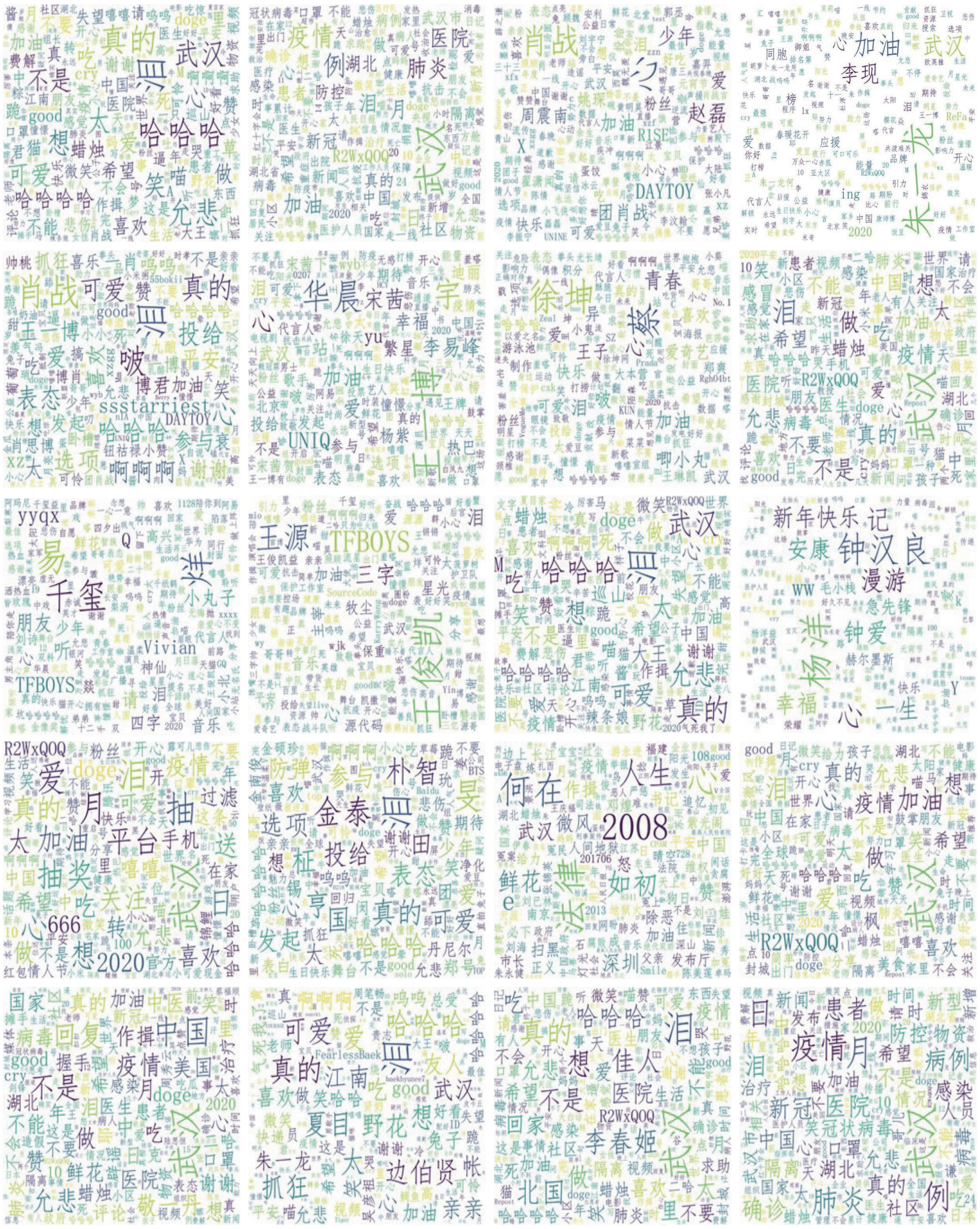

Figure S7. Word cloud of each community in the severe stage.

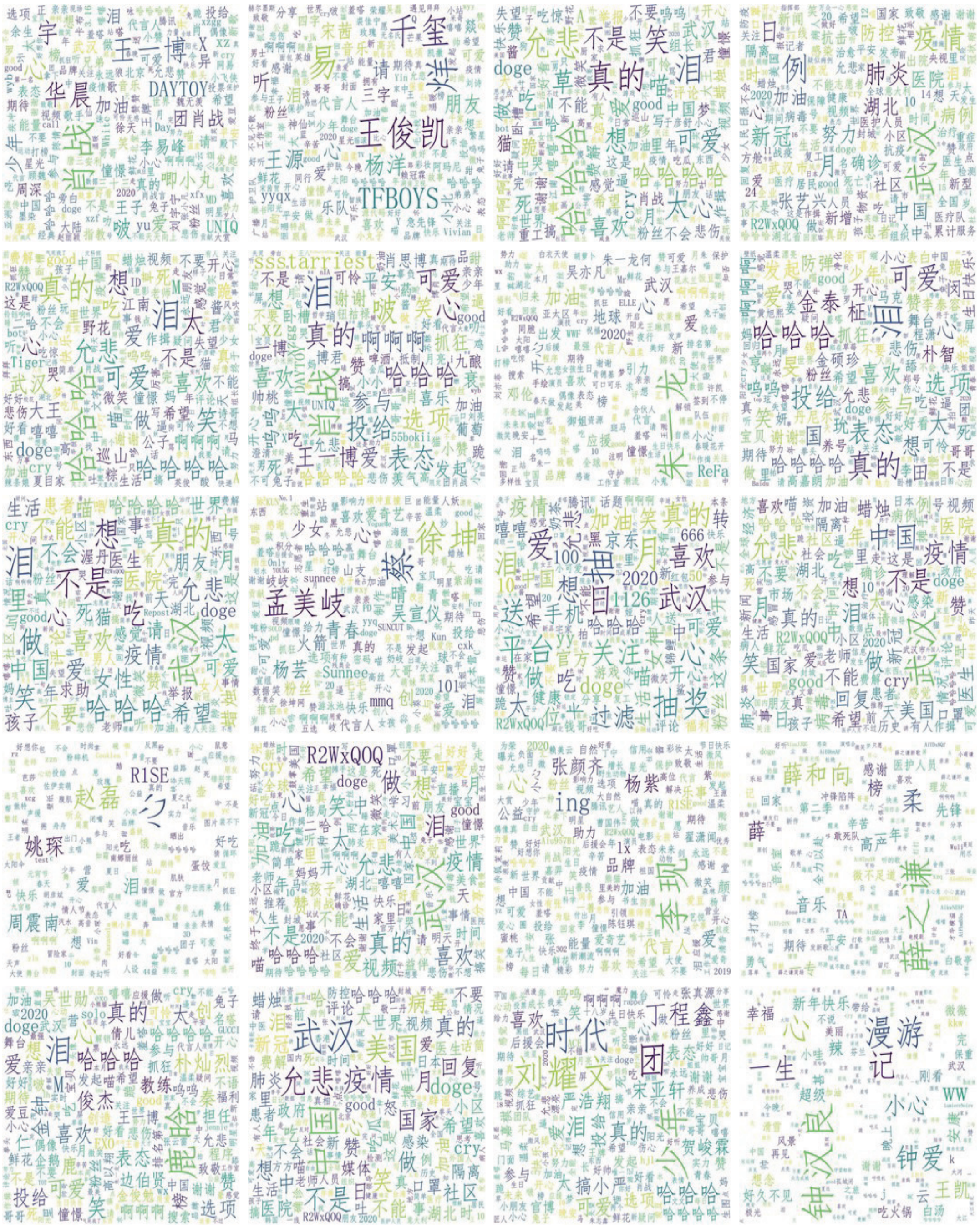

Figure S8. Word cloud of each community in the recovery stage.
